# Gitelman syndrome with hypercalcemia and normomagnesemia: A case report

**DOI:** 10.1097/MD.0000000000042610

**Published:** 2025-05-30

**Authors:** Zhenlin Tan, Chen Liu, Zheng Feng, Zhimei Luo, Xiaofen Lian, Donghui Lu

**Affiliations:** a Department of Endocrinology, Peking University Shenzhen Hospital, Shenzhen, Guangdong, China; b Shantou University Medical College, Shantou, Guangdong, China.

**Keywords:** Gitelman syndrome, hypercalcemia, point mutation

## Abstract

**Rationale:**

Gitelman syndrome (GS) is a rare autosomal recessive renal tubular disease, whose main symptoms are long-term hypokalemia, hypomagnesemia, hypochloremic metabolic alkalosis, and hypocalciuria.

**Patient concerns:**

This study reported a GS patient with hypercalcemia combined with normomagnesemia.

**Diagnoses:**

GS with hypercalcemia.

**Interventions:**

The patient was treated with a 20 mg spironolactone tablet 2 times/day and a 1 g potassium chloride sustained-release tablet 3 times/day for potassium preservation.

**Outcomes:**

The patient received regular individualized long-term potassium supplementation and was followed regularly.

**Lessons:**

GS is an extremely rare disease, which is characterize by long-term hypokalemia, hypomagnesemia, hypochloremic metabolic alkalosis, and hypocalciuria. But the case reported here combined with hypercalcemia and normomagnesemia. Clinical physicians should increase their awareness of this disease to enable early diagnosis and treatment of GS and reduce missed diagnoses.

## 1. Introduction

Gitelman syndrome (GS) is a rare autosomal recessive renal tubular disease. Hypocalciuria, hypochloremia, and metabolic alkalosis were the main clinical features. The pathogenesis of GS is due to the mutation of the SLC12A3 gene on chromosome 16q13, which encodes a thiazide diuretic sensitive sodium chloride cotransporter (NCC) located in the apical membrane of the epithelial membrane of DCT of the kidney. This in turn causes increased excretion of urinary electrolytes, including potassium ions, hydrogen ions, and magnesium ions, while reducing urinary calcium excretion.^[1]^ Common clinical symptoms of GS patients can manifest in multiple systems of the body, such as the kidney, gastrointestinal tract, skeletal muscle, cardiovascular system, and nervous system.^[2]^ This paper reports a case of clinically suspected GS with hypercalcemia and normomagnesemia. Whole-exome gene sequencing analysis was performed for a patient in the pedigree, which provides a clinical theoretical basis for the clinical diagnosis and subsequent treatment of GS, as well as guidance for early genetic screening, prevention, diagnosis and treatment of the family members.

## 2. Case presentation

A 58-year-old man was admitted to the Department of Endocrinology, Peking University-Shenzhen Hospital, in July 2023 due to “recurrent hypokalemia for 12 years and another month.” The patient had no obvious cause of hypokalemia since 2011 and was hospitalized in other hospitals many times. The patient regularly received potassium chloride sustained-release tablets for potassium supplementation. The patient had seen a doctor in our department 1 month prior. The potassium test result was 2.96 mmol/L, and the patient was treated with potassium supplementation (1.0 g) via a potassium chloride sustained-release tablet twice a day. The patient had a previous history of low blood pressure, hypercalcemia, hyperuricemia, hyperlipidemia, adrenal enlargement, and bilateral renal cysts. He had a blood pressure of 98/85 mm Hg (1 mm Hg = 0.133 kPa), and the other physical examination revealed no specific findings.

Laboratory Tests: Routine blood tests revealed the following: white blood cell count was 6.75 × 109/L, while hemoglobin was 148g/L and platelets were 340 × 109/L. Routine urine examination revealed a pH of 7.0 and a urine specific gravity of 1.020, and microscopic examination of the cells and casts was negative. Blood biochemistry revealed the following: total protein was 71.5 g/L; Albumin was 42.0 g/L; Alanine transaminase was 23 U/L; Total bilirubin was 15.4 µmol/L; creatinine was 83 µmol/L; uric acid was 554 µmol/L; glomerular filtration rate was 87.1 mL/min; total cholesterol was 4.00 mmol/L; triglyceride was 0.87 mmol/L; fasting plasma glucose was 5.26 mmol/L; hemoglobin A1c level was 5.8%; blood potassium was 2.90 mmol/L; blood sodium was 140 mmol/L; blood chloride was 98.2 mmol/L; blood calcium was 2.54 mmol/L; and blood magnesium was 0.69 mmol/L. Blood gas analysis revealed the following parameters: pH 7.480, bicarbonate concentration 28.3 mmol/L, partial pressure of carbon dioxide 38.0 mm Hg, and partial pressure of oxygen 71 mm Hg. There were 4 items related to hypertension (upright position): renin activity, 7.52 ng/mL/h; aldosterone, 175.21 pg/mL; angiotensin I, 9.15 pg/mL; and angiotensin II, 182.66 pg/mL. His blood catecholamine levels were 36.10 pg/mL for epinephrine, 312.00 pg/mL for norepinephrine, and 4.71 pg/mL for dopamine. His blood parathyroid hormone level was 3.70 pmol/L. His serum calcitonin concentration was 2.45 pg/mL. Cortisol, adrenocorticotropic hormone, sex hormones, Insulin-like growth factor-1, the growth hormone concentration, 24-hour urine aldosterone level was within the normal range. A plain pituitary magnetic resonance imaging scan showed that the left pituitary gland was slightly fuller than the contralateral side. Adrenal gland computed tomography imaging with non-contrast, contrast-enhanced, and 3D reconstruction protocols revealed that the left adrenal trunk and lateral limb were slightly thicker, with small nodules in the right adrenal trunk and cysts in both kidneys. Color Doppler ultrasound examination of both kidneys revealed a cystic space-occupying lesion in the left kidney, and a renal cyst was considered.

**Figure 1.**
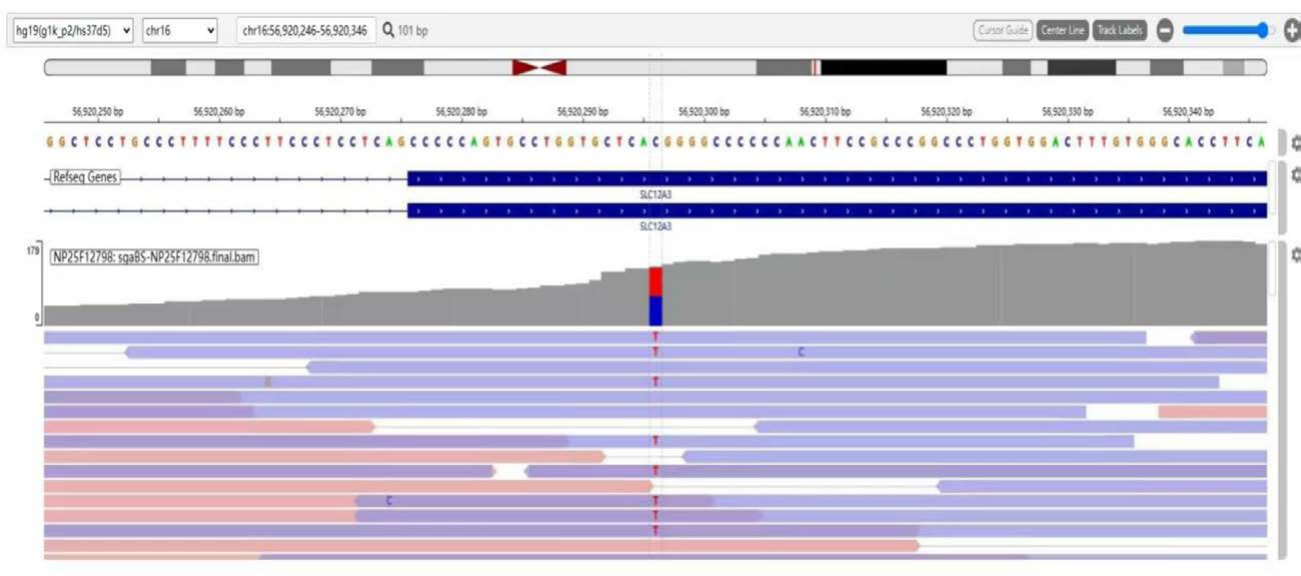
The sequencing peak map.

Genetic testing: The peripheral blood collected from the patient was sent to Guangzhou KingMedical Medical Laboratory Center Co., Ltd., Guangzhou for the detection of relevant disease-causing genes. The results of the patient’s blood electrolytes and 24-hour simultaneous urine electrolyte test and genetic detection results are shown in Tables 1 to 3, respectively. The biochemical examination results of the patient during hospitalization revealed hypokalemia, hypochloremia, hypercalcemia, metabolic alkalosis, high activity of the renin-angiotensin-aldosterone system, normal blood magnesium, and normotension. The results of serum electrolytes and synchronous 24-hour urine electrolytes further suggested that potassium loss was caused by renal activity. A whole-exome gene sequencing revealed 2 heterozygous missense mutations in the coding region of the SLC12A3 gene (OMIM: 263800), i.e., cytosine C was changed to thymine at nucleotide 961 (c.961C > T), causing arginine no. 321 to change to tryptophan (p.Arg321Trp), another heterozygous missense mutation, i.e., a change from cytosine C to thymine T at nucleotide 19426 (c.19426C > T), resulting in a change from threonine 649 to methionine (p. Thr649Met). The sequencing peak map shows in Figure 1. However, the mutation site c.961C > T has been previously reported, but according to the existing evidence, the clinical significance of this locus is unclear. Another mutation locus, c.19426C > T, has also been reported in the literature, and the existing evidence indicates that this locus is associated with GS and has pathogenic variants. Therefore, based on the biochemical and SLC12A3 gene detection results, the final diagnosis was GS with a compound heterozygous mutation of hypercalcemia combined with normomagnesemia. During hospitalization, biochemical examination did not reveal low blood magnesium. Therefore, the patient was given only oral potassium chloride sustained-release tablets for potassium supplementation. However, the increase after potassium supplementation was not significant. Then, the patient’s blood potassium gradually increased after the addition of 40 mg spironolactone (aldosterone antagonist) (spironolactone), but the serum potassium was still lower than the lower limit of the normal value. The patient’s serum potassium level was 2.87 to 3.05 mmol/L before discharge and he was followed after discharge. Currently, he is being treated with a 20 mg spironolactone tablet 2 times/day and a 1 g potassium chloride sustained-release tablet 3 times/day. The blood electrolytes were monitored.

**Table 1:** Results of blood electrolytes (mmol/L).

Date	Potassium	Chloride	Calcium	Magnesium
2023.07.31	3.05 (3.5–5.3)	99.4 (90–110)	2.58 (2.11–2.52)	0.73 (0.75–1.02)
2023.08.01	2.87 (3.5–5.3)	99.8 (90–110)	2.53 (2.11–2.52)	0.73 (0.75–1.02)
2023.08.04	2.95 (3.5–5.3)	98.9 (90–110)	2.48 (2.11–2.52)	0.72 (0.75–1.02)
2023.08.31	2.87 (3.5–5.3)	99.8 (90–110)	2.53 (2.11–2.52)	0.73 (0.75–1.02)
2023.10.24	2.91 (3.5–5.3)	97.5 (90–110)	2.76 (2.11–2.52)	0.88 (0.75–1.02)
2024.03.29	2.72 (3.5–5.3)	97.1 (90–110)	2.61 (2.11–2.52)	0.82 (0.75–1.02)
2024.07.29	3.02 (3.5–5.3)	98.3 (90–110)	2.69 (2.11–2.52)	0.76 (0.75–1.02)
2024.08.23	3.80 (3.5–5.3)	99.4 (90–110)	2.89 (2.11–2.52)	N

N = no detected.

**Table 2:** Results of 24-hour urine electrolyte test (mmol/L).

Date	Potassium	Chloride	Calcium	Magnesium
2023.07.28	101.5 (25–100)	210 (140–250)	2.2 (2.2–7.5)	3.25 (3–5)
2023.07.31	80.70 (25–100)	224 (140–250)	1.94 (2.2–7.5)	3.40 (3–5)
2023.08.01	100.1 (25–100)	297 (140–250)	2.38 (2.2–7.5)	4.48 (3–5)

**Table 3:** Results of genetic testing.

Gene chromosomal location	Transcription exons	Nucleotides	Amino acid	Protein levels	Inheritance	Pathogenicity
SLC12A3	chr16:56920296	NM_001126108.2	c.1946C > T	p.Thr649Met	AR	Pathogenicity
SLC12A3	chr16:56906371	NM_001126108.2	c.961C > T	p.Arg321Trp	AR	Unknown clinical significance

## 3. Discussion

GS, also known as familial hypokalemic and hypomagnesemia, is a renal tubular disease that is different from other common renal tubular diseases.^[3]^ This case reported occurred in adulthood, and the clinical symptoms were atypical. Laboratory tests revealed low blood potassium, high blood calcium, hypochlorite metabolic alkalosis, and hypocalciuria, while blood magnesium was normal. The screening test for the diagnosis of GS is the hydrochlorothiazide test.^[4]^ In respect of the patient’s choice, we proceeded directly to the genetic testing without performing the hydrochlorothiazide test. Currently, hypercalcemia with normal blood magnesium GS has not been reported in the literature. GS should be differentiated from Bartter syndrome. The 2 have similar clinical features and biochemical parameters. However, the key points for distinguishing between the 2 are mainly age of onset, low urine calcium and low blood magnesium. The mutation locus found through genetic testing is the main basis for diagnosis. The genetic testing revealed 2 heterozygous missense mutations in SLC12A3 gene, the diagnosis of GS was confirmed.^[4,5]^ In addition, adrenal enlargement of the patient’s left kidney was detected. Cysts in both kidneys were differentiated from hypokalemia caused by primary aldosteronism, Cushing syndrome, and congenital adrenal hyperplasia. Combined with the lack of corresponding clinical manifestations and normal hormone levels, the cyst was considered to have no functional changes, but long-term follow-up observation is still needed.

Currently, more than 500 mutations in the SLC12A3 gene that can cause GS, such as splicing mutations, missense mutations, and nonsense mutations. The most common mutations are missense mutations and nonsense mutations.^[6]^ Gene mutation loci vary between countries or regions. In Europe, the most common mutation loci are p.Gly741Arg, p.Cys994Tyr, p.Leu859Pro, and p.Arg861Cys.^[7]^ However, in China, the frequency of SLC12A3 gene mutations is high, and the most common mutations are p.T60M and p.D486N.^[8–16]^ In theory, only the homozygous mutation is needed to cause the pathogenesis of GS, but in clinical practice, there are also patients with partial compound heterozygous mutations and simple heterozygous mutations. 2 point mutations were found in this patient: c.961C > T (p.Arg321Trp) and c.1946C > T (p.Thr649Met). Although these 2 mutation loci have been reported in the literature,^[17]^ due to the low frequency in our current reference population gene database, compound heterozygous mutations in these 2 mutation loci have not been reported. Notably, c.1946C > T (p.Thr649Met) was reported as a pathogenic variant,^[17]^ while the mutation locus c.96 1C > T. However, the clinical significance of the mutation site c.961C > T (p.Arg321Trp) was suggested to be of unclear clinical significance in the patients in this study.

This patient sought medical treatment many times due to refractory hypokalemia and had hypercalcemia, normal blood magnesium, multiple measurements of serum potassium < 3.5 mmol/L, and blood calcium > 2.52 mmol/L, while serum potassium was < 3.5 mmol/L, urinary potassium excretion was > 25 mmol/24 h. At the same time, the patient had hypocalciuria, and the random urine calcium/urine creatinine ratio was < 0.2 mmol/mmol. Based on the 2021 Chinese Expert Consensus, which classifies the severity of GS according to serum potassium levels, serum magnesium levels, and clinical manifestations,^[16]^ we categorized this patient’s hypokalemia severity as Grade 2 and clinical manifestations as Class A. The specific grading criteria are summarized in Tables 4 and 5. Three 24-hour urine calcium measurements were performed. All 3 measurements of urine calcium were < 2.5 mmol/L/24 h. The urinary calcium excretion characteristics were consistent with the description in the guidelines.^[4]^ In addition, the calculated chloride ion excretion fraction was > 0.5%, the plasma renin activity (supine position) was 2.62 ng/mL, and the patient’s blood pressure was normotensive. Due to the inability to obtain peripheral blood samples from the patient’s parents and children, we could only perform long-term follow-up of the patient and monitor the relevant biochemical indicators.

**Table 4:** Severity classification of hypokalemia and hypomagnesemia in patients with Gitelman syndrome (GS).

Grade	1	2	3	4
Serum K⁺ (mmol/L)	3.0–3.4	2.5–2.9	2.0–2.4, *or* requiring intensive replacement therapy/hospitalization	<2.0, *or* hypokalemia with paralysis/intestinal obstruction, *or* life-threatening arrhythmias
Serum Mg²⁺ (mmol/L)	0.60–0.70	0.45–0.59	0.30–0.44	<0.30, *or* associated with life-threatening arrhythmias/tetany

**Table 5:** Grading recommendations for clinical manifestations of Gitelman syndrome (GS).

Grade	Clinical manifestations
A	Asymptomatic hypokalemia (incidental detection via laboratory tests) or mild symptoms (e.g., fatigue, polydipsia, polyuria) without impacting daily life or causing distress.
B	Noticeable symptoms that moderately impair quality of daily life.
C	Severe or medically significant manifestations (nonlife-threatening), requiring hospitalization or prolonging hospital stay; severely impairing daily life and may impair self-care ability.
D	Life-threatening conditions requiring emergency intervention.

Currently, there is no cure for GS patients. The main treatment goals for GS patients are to effectively improve their symptoms to further improve their quality of life while maintaining a balance of electrolytes in the body. The most important treatment program is individualized long-term potassium supplementation. When combined with low blood magnesium, magnesium supplementation should be considered a priority. This not only helps to improve hypokalemia^[18]^ but can also improve other symptoms, such as neuromuscular symptoms and depressive status.^[16,19]^ After the patient was given potassium supplementation treatment with potassium chloride sustained-release tablets, his blood potassium level improved significantly. According to a previous review, his blood potassium level was 2.95 mmol/L, which is close to the target value. After discharge from the hospital, the patient was treated with potassium chloride sustained-release tablets and spironolactone for a long time.

In most cases of GS, the underlying pathological mechanism involves mutations in the SLC12A3 gene, leading to dysfunction of the NCC in the distal convoluted tubule (DCT).^[3]^ This results in reduced reabsorption of sodium and chloride in the DCT, with subsequent urinary loss of these ions. Consequently, this leads to the characteristic manifestations of hypokalemia, hypomagnesemia, hypochloremic metabolic alkalosis and hypocalciuria. Among these, NCC dysfunction and reduced transient receptor potential channel melastatin subtype 6 expression are the primary pathogenic mechanisms underlying hypomagnesemia.^[20]^ However, in this case, the patient exhibited normal serum magnesium and urinary magnesium levels. We reasonably hypothesize that the missense mutation in the coding region of the SLC12A3 gene may only partially impair the NCC function, thereby preserving partial sodium and chloride reabsorption capacity. This residual activity could attenuate magnesium reabsorption impairment in DCT. GS is characterized by hypocalciuria and hypomagnesemia, and it does not directly lead to hypercalcemia. The occurrence of hypercalcemia is generally associated with secondary hyperparathyroidism or comorbid disorders. However, in this case, the parathyroid hormone level was within the normal range, and no evidence of malignancy or functional adenoma was identified. The exact etiology of hypercalcemia remains undetermined.

In summary, early identification, diagnosis and intervention are particularly important for GS. Clinically, GS patients often exhibit metabolic alkalosis, such as hypokalemia, low blood magnesium, high urine potassium, low blood chloride, and normal or low blood pressure. Due to the diverse clinical manifestations of GS, it is particularly important to perform genetic testing on patients and their families with suspected GS. GS can present with different electrolyte imbalances. Therefore, we suggest clinicians pay close attention to patient electrolytes. Active treatment, scientific management, and regular follow-up are necessary for patients.

## Acknowledgments

We acknowledge the patient and his family for their support of this manuscript.

## Author contributions

**Conceptualization:** Zhimei Luo.

**Data curation:** Zheng Feng.

**Formal analysis:** Xiaofen Lian.

**Supervision:** Donghui Lu.

**Writing – original draft:** Zhenlin Tan, Chen Liu.

**Writing – review & editing:** Chen Liu.
